# Young people with depression and their experience accessing an enhanced primary care service for youth with emerging mental health problems: a qualitative study

**DOI:** 10.1186/1471-244X-12-96

**Published:** 2012-08-01

**Authors:** Terence V McCann, Dan I Lubman

**Affiliations:** 1 School of Nursing and Midwifery, Victoria University, PO Box 14428, Melbourne, 8001, VIC, Australia; 2 Turning Point Alcohol and Drug Centre, Eastern Health and Monash University, 54-62 Gertrude Street, Fitzroy, 3065, VIC, Australia

**Keywords:** Depression, Early intervention, Help-seeking, Interpretative phenomenological analysis, Qualitative research, Service access, Youth mental health, *Headspace*

## Abstract

**Background:**

Despite the emergence of mental health problems during adolescence and early adulthood, many young people encounter difficulties accessing appropriate services. In response to this gap, the Australian Government recently established new enhanced primary care services (*headspace*) that target young people with emerging mental health problems. In this study, we examine the experience of young people with depression accessing one of these services, with a focus on understanding how they access the service and the difficulties they encounter in the process.

**Method:**

Individual, in-depth, audio-recorded interviews were used to collect data. Twenty-six young people with depression were recruited from a *headspace* site in Melbourne, Australia. Interpretative phenomenological analysis was used to analyse the data.

**Results:**

Four overlapping themes were identified in the data. First, school counsellors as access mediators, highlights the prominent role school counsellors have in facilitating student access to the service*.* Second, location as an access facilitator and inhibitor. Although the service is accessible by public transport, it is less so to those who do not live near public transport. Third, encountering barriers accessing the service initially. Two main service access barriers were experienced: unfamiliarity with the service, and delays in obtaining initial appointments for ongoing therapy. Finally, the service’s funding model acts as an access facilitator and barrier. While the model provides a low or no cost services initially, it limits the number of funded sessions, and this can be problematic.

**Conclusions:**

Young people have contrasting experiences accessing the service. School counsellors have an influential role in facilitating access, and its close proximity to public transport enhances access. The service needs to become more prominent in young people’s consciousness, while the appointment system would benefit from providing more timely appointments with therapists. The service’s funding model is important in enabling access initially to young people from low socioeconomic backgrounds, but the government needs to reassess the model for those who require additional support.

## Background

According to the 2007 *National Survey of Mental Health and Wellbeing*[1], more than one in four Australians aged 16–24 years had experienced a mental disorder (most commonly affective, anxiety and/or substance use disorders) in the previous 12 months. Affective disorders were identified as the diagnosis associated with the greatest severity, with more than one in four experiencing symptoms of, and 6.3% being diagnosed with, depression. However, fewer than 25% of young people with mental health problems sought professional help [2], which is concerning given delays in accessing treatment can significantly affect social, educational and vocational outcomes, and may have adverse long-term consequences [3].

Young people with depression can encounter considerable personal and logistical barriers when accessing services. Personal barriers, such as embarrassment [4] and hoping the problem will go away [5], are considered more problematic than logistical barriers, such as difficulties in getting appointments and obtaining transport, and the cost of services [4,6,7]. Nevertheless, access to services remains an important factor when examining pathways to care, together with a service’s ability to identify and respond appropriately to symptoms [8].

Many young people do not have easy access to, or are reluctant to approach, primary care services [9]. Factors that contribute to delays in help-seeking are community stigma of youth depression [10,11] and stigmatising attitudes of mental health professionals [12], both of which lead to a longer duration of untreated illness (DUI) [13]. As a result, many young people feel embarrassed and self-consciousness about approaching professionals for help, perceiving that others will react negatively to them if they seek treatment for depression [4,14]. Another contributor is that young people are reluctant to consult with general practitioners (GPs) [4], which in turn, adversely affects the likelihood of referral to mental health services. It is claimed that youth-friendly models of care, where positive engagement occurs so youth feel valued, respected and supported to take control of their lives [15], are less likely to be stigmatised and are more accessible than adult service models [9,16], where initial access and ongoing involvement is difficult and contributes to DUI [13]. However, a lack of available youth-friendly models prevent youth with significant morbidity and related functional impairment from obtaining appropriate assistance [17-19]. It is essential, therefore, that there is an investment in developing youth-friendly models of care within primary care settings.

In response to this need, the Australian Government established *headspace,* the National Youth Mental Health Foundation, in 2006. This initiative aims to increase young people’s access to services and reduce disease burden attributable to mild-to-moderate mental health and substance use issues by promoting improvement in their mental health, social wellbeing and economic participation [9,20]. As an evolving model of enhanced primary care for young people, it seeks to do this by providing holistic services, including physical health assessment and treatment; increasing community capacity to identify young people with mental health problems early; supporting help-seeking by young people and their carers; providing evidence-based interventions; and improving service integration through co-location with other services [9,20]. Other features of the service are its multidisciplinary approach comprising, for example, GPs, psychiatrists, and nursing and allied health clinicians; co-location with other government and non-government services for young people; and a mixed model of funding consisting of centralised infrastructure support and outpatient services that attract some level of government and private health insurance rebate. Currently, there are over 30 *headspace* sites in metropolitan, regional and remote areas of Australia.

Even though this enhanced primary care service for youth with emerging mental health issues is a significant development, limited research has been conducted examining young people’s experience of accessing this type of treatment model, nor identifying which access barriers remain problematic and need to be addressed. This is particularly important as a large proportion of young people remain reluctant to seek help for mental health problems such as depression [2,4,14]. While this issue has been comparatively well documented in older adults, much less is known about the help-seeking experience of young people in Australia and internationally. Moreover, few studies have incorporated a qualitative paradigm, despite the importance of this approach for providing a rich insight into under-researched areas. This may be attributable to greater emphasis being placed on the use of quantitative paradigms, but such approaches are less suited to providing an in-depth exploration of issues.

In this study, we examine the experience of young people with depression accessing a new enhanced primary care service for youth, with an emphasis on understanding how they access the service and the barriers they encounter in the process.

## Method

Interpretative phenomenological analysis (IPA), a hermeneutic or interpretative method founded primarily on the Heideggerian view of phenomenology [21], was used to guide data collection and analysis. The key features of IPA are phenomenology, hermeneutics and idiography [22]. The method is phenomenological because of the focus on understanding how young people make sense of their major life experiences, in this instance their experience of accessing a new type of enhanced primary care service that focuses on youth mental health issues. IPA is regarded as an interpretative method informed by hermeneutics, the theory of interpretation [23]. The approach is idiographic because of the focus on commencing with the young person with depression as the unit of analysis and then progressing gradually to develop themes and subthemes [24]. The interplay between inductive and deductive procedures in IPA means that as the analysis progresses existing theory can be endorsed, modified, and/or challenged. Finally, the method is particularly useful where the problem is new or under-researched, where issues are diverse or unclear, and where the researcher seeks to understand process and change [25].

### Participants

Young people with a primary diagnosis of depression were recruited through clinician referral, from a single *headspace* service within northwestern Melbourne. Purposive or criterion sampling was used to inform data collection [26,27]. Inclusion criteria were: (i) primary diagnosis of depression, and (ii) aged 16–25 years. Exclusion criteria were (i) history of psychosis, (ii) currently expressing suicidal plans, and (iii) unable to communicate in conversational English.

Interviews proceeded until no new themes were identified in the data. Furthermore, saturation of identified themes with ‘thick’ description of the data was obtained when no new data emerged to support these themes. Each theme contained thick descriptions; deep, dense, depictions [28,29] of the young people’s experiences accessing the service. It was this process that determined the actual number of participants in the study, an important part of the rigour of the qualitative approach to determining sample size [30,31].

### Procedure

Ethical approval was obtained from Victoria University Human Research Ethics Committee and Melbourne Health Human Research and Ethics Committee. All participants gave informed consent, including written parental/guardian consent when aged under 18 years. Data collection took place in a private room at the service. Semi-structured, audio-recorded interviews were carried out, each lasting 30 to 60 minutes. Broad questions were asked initially, such as “Can you tell me the good things, if any, about your experience accessing the service?” “Can you tell me the difficulties, if any, about accessing the service?” Can you tell me the changes that are needed, if any, to improve your access to the service? Responses to questions were probed and explored further. At the conclusion of each main part of the interview, the researcher summarised the content to ensure the participant’s perspective was stated and comprehended correctly, a verification process that enhanced the credibility of the study [32].

### Data analysis

A six-step process was used to carry out a thematic analysis of the interview transcripts, consistent with Smith and Osborn’s [23] guidelines for adopting the IPA method. (i) Transcribed data were read and re-read to obtain a broad appreciation of young people’s experience accessing the service. (ii) Manual coding of the raw data, using in vivo codes [33], was undertaken. These types of codes relate directly to the words used by the participants, and help avoid situations where researchers may otherwise impose their preconceived frameworks and opinions on the data [33,34]. (iii) Codes were grouped together, and from these, themes were identified. (iv) The latter were then clustered into groups of themes and sub-themes. (v) At the same time, data reduction took place with identified themes insufficiently grounded in the data being omitted. (vi) A more intense analytical arranging of themes then occurred. This comprised rearranging and refining themes and abstracting them to a higher level [23]. Finally, Hill, Thompson & Williams’ [35] criteria were used to determine the representativeness of themes: ‘general’ — applied to all cases; ‘typical’ — related to half or more cases; ‘variant’ — applied to more than two but less than half the cases; and those that relate to only one or two cases were not reported.

### Rigour

The methodological rigour of the study was established in four ways. Dependability and confirmability were established by developing an audit trail to link raw data and codes with themes, and verification from participants [26,32]. In addition, coding and thematic analysis was carried out by one of the authors, followed by an independent review of the process by another researcher. Credibility was assured by using a semi-structured interview guide to maintain a consistent way of interviewing. Credibility was also reinforced by participant verification, which entailed summarising the young people’s comments to ensure that they were understood correctly [26,33]. Transferability was maintained by presenting sufficient data in this paper to give readers the opportunity to evaluate the findings and to determine their transferability to other situations. Overall, adhering to this trustworthy approach ensured that the themes developed in the context of the young people’s experience accessing the service could be transferred to other similar programs [33].

## Results

Thirty-two young people were invited to take part in the study, and of these, 26 consented to participate (no one subsequently withdrew). Participants’ ages ranged from 16–22 years, with a mean age of 18 years (Table 1). Sixteen were young women, most were single, and fifteen lived in the same household as one or both parents. Six were still attending high school, and seven were engaged in various forms of paid employment. In order of frequency, their main reasons for attending the service were for treatment of depression and anxiety, depression, and depression and comorbid substance use (predominantly alcohol, cannabis and amphetamines) respectively. Their average length of engagement with the service was less than five months.

**Table 1:** Sociodemographic details of participants

**Variables**	**n = 26 (%)**
Gender	
Young women	16 (61.5 %)
Young men	10 (38.5 %)
Marital status	
Single	19 (73.1 %)
Married/de facto	4 (15.4 %)
Boyfriend/girlfriend	2 (7.6 %)
Missing data	1 (3.8 %)
Living circumstances	
Lives with one or both parents	15 (57.7 %)
Lives with spouse/partner	1 (3.8 %)
Lives with boyfriend/girlfriend	1 (3.8 %)
Lives with other relatives	2 (7.7 %)
Lives alone in non-supported accommodation	2 (7.7 %)
Lives alone in supported accommodation	1 (3.8 %)
Homeless	1 (3.8 %)
Other†	3 (11.5 %)
Highest level of education completed	
High school (Yr. 10)	12 (46.2 %)
High school (Yr. 12)	7 (26.9 %)
High school (continuing)‡	6 (23.1 %)
University	1 (3.8 %)
Paid employment	
Yes	7 (26.9 %)
No	19 (73.1 %)
Employment type	
Full-time	1 (3.8 %)
Part-time	4 (15.4 %)
Casual	2 (7.7 %)
	Mean (SD)
Age	18 (1.8 years) (Range 16–22 years)
	Median (SD)
Duration of contact with the service	4.5 (5.1 months) (Range 0–24 months)

† = living with foster parent, parents and partner, stepfather.

‡ = still attending high school.

Four overlapping themes were identified in the data, reflecting the experience of young people accessing the enhanced primary care service: (i) school counsellors as service access mediators, (ii) service location as an access facilitator and inhibitor, (iii) encountering barriers accessing the service initially, and (iv) service funding as an access facilitator and barrier.

### School counsellors as service access mediators

In this typical theme, young people who were attending school acknowledged the important role that supportive school counsellors played in facilitating access to the youth service. These participants had easy access to school counsellors, who in turn, made arrangements for the young people to get time off school, if necessary, to attend the youth service. School counsellors, in particular those who were familiar with the existence and the benefits of accessing this type of enhanced primary care facility, provided a key link between the young person and the service. Likewise, the existence of a trusting relationship between the school counsellor and the young person increased the likelihood of the latter accessing the service.

"Well I, okay, I ended up at *headspace* in a kind of normal way, I guess; like, I went to my school counsellor and my school counsellor made me call up *headspace* (Interviewee 3)."

"It was, like, pretty much organised for me, and so that made it so much easier for me. And, like, it was really accessible, and it was really available to me because I was referred to *headspace* by my school counsellor (Interviewee 22)."

While, overall, supportive school counsellors were regarded an important means of referral to the service, some young participants were reluctant to approach these staff because of the perception of possible breaches of confidentiality by the counsellor. This meant that the young person was unable to be referred to, or become aware of, the youth service through this route.

"Well, school counselling, I didn’t really like it that much, because, like, the school counsellor saw friends of mine that needed counselling; and it was just messy ‘cause they would have been talking about me or sort of those things (Interviewee 5)."

### Service location as an access facilitator and inhibitor

In this general theme, the location of the youth service influenced access to the facility. Two contrasting sub-themes about geographical accessibility were identified. The service was easily accessible by public transport; two railway stations were located within walking distance, and bus services were even closer.

"Well, it’s [youth service] near the train station and I take the train every single day to go to school. And, so after school I’ll just take a train to … [name of suburb where the service is located], get off and walk, like, some five minutes and I’m here (Interviewee 25)."

Alternatively, even though there was public transport nearby, the service was less accessible to those whose homes were not in close proximity to public transport, did not have their own transport, or were reliant on others to bring them to the service. For some, if they did not have someone to bring them to the service then they might not have accessed the service: *“… it would be pretty difficult if my mum didn’t take me ‘cause I live in [name of suburb], sort of like half an hour’s drive. But if she didn’t take me I wouldn’t come really* (Interviewee 21).

Furthermore, relying on others to overcome difficulties with geographical access to the service sometimes caused problems within the home and exacerbated the young person’s already depressed state.

"It’s a little far away from my school and quite far away from where I work. So a lot of the time if I can’t get public transport, because it takes so long and I might be running late, my mum has to drive me, and it’s just like difficult for her sometimes because sometimes she’s cooking, or cleaning the house, or looking after my little brother. And a lot of times that leads to fights [arguments] and those fights lead to me being depressed (Interviewee 26)."

### Encountering barriers accessing the service initially

In this variant theme, two main barriers were encountered accessing the youth service; unfamiliarity with the service, and delays in obtaining initial appointments. Unfamiliarity stemmed from a lack of knowledge of the existence of the service. Although a few young people reported that they were aware of the service’s whereabouts prior to help-seeking, others were unaware of its existence. This was more likely to be the case for those who were reluctant to approach school counsellors or no longer attended school. In such circumstances, access pathways were more wide-ranging and less assured, including self-referral or recommendations from friends and GPs who were aware of the existence of the service. In these situations, help-seeking was dependent on the young person’s or others’ prior knowledge of the service, but this was not always the case, as illustrated in the following two exemplars:

"Basically, … putting the word out and all that, ‘cause not many people know about the place [*headspace*], so I reckon if they had more ways of getting young people like my age … to come use it (Interviewee 11)."

"I’ve never heard anything about it before my school counsellor, so, if anything, advertise it a little bit more. (Interviewee 17)."

Another perceived access barrier was that when participants approached the service initially, they commonly encountered delays getting timely appointments with clinicians providing ongoing therapy, particularly clinical psychologists. However, once an initial appointment was gained, subsequent access was much easier.

"The waiting list; the first, the initial waiting list. Once you’re in, you can keep coming on a regular basis but to get in in the first place, there’s like a month waiting. I booked in my appointment in June, [but] I didn’t come until … [late] August; so it’s a month and a half (Interviewee 4)."

An additional perceived access barrier was that, in some circumstances, due to increased demands on the service, appointments had to prioritised, based on the assessed level of need. Those whose needs were assessed by the service’s clinicians as more acute and urgent were given higher priority over those assessed as requiring less immediate attention: *“Like you get on a waiting list to get help and it takes ages; or it’s like, ‘sorry, you’re not kind of sick enough for us to look after you’”* (Interviewee 4). The implication of prioritising was that for those designated as in less immediate need of assistance, it was a frustrating wait and might have deterred some from persevering with their appointment.

### Service funding as an access facilitator and barrier

In this variant theme, the issue of whether youth are required to pay for treatment is an important consideration. Young people did not incur any out-of-pocket fees or charges when they accessed the youth service initially. This arrangement was made through Medicare, the Australian Government’s universal public health care system [36], which approved a fee that the service provider could charge at each consultation, or alternatively through private health insurance reimbursement. This approach facilitated access to treatment, particularly to those from low socioeconomic backgrounds: *“headspace is okay, especially for people like me, low income earners …”* (Interviewee 4). However, there was a limit (12 at the time of the study) on the number of consultations for individual therapy that the young person could receive through this scheme in a single calendar year. In essence, for those who required additional therapy and had limited financial resources, this limited ongoing access to the service. A possible consequence of this funding limitation was it might have deterred some young people from continuing their engagement with the service.

"Under Medicare you only get 12 sessions free. So who’s to say that you’re going to be cured in 12 sessions? And what happens for a young person if after the 12 sessions they still need help and they can’t afford to pay for a session? (Interviewee 2)."

## Discussion

This exploratory study provides a first look at understanding the experiences of young people with depression accessing a new primary care initiative aimed at increasing treatment access for young people with mental health issues. We present four overlapping themes that depict the facilitators and barriers that they encounter in the process. First, they utilise several access pathways to the service. For those still attending high school they were referred frequently to the service by school counsellors. Clearly, counsellors have an important role in making a link with the service. It highlights that they have developed a trusting relationship with the young person, and are knowledgeable of the existence of the service and perceive it as helpful to young people. However, for youth reluctant to approach school counsellors, or no longer attending school, the access pathway to the service is more tortuous and uncertain. This is chiefly the case for those who are reluctant initially to seek professional help through formal access pathways, such as counsellors and GPs [3,9]. They are more likely to be dependent on trusted, informal access pathways, such as family and friends, before attempting to access formal services [3,9], because few consider GPs as their preferred means of help [4].

Second, another dimension of access to the service is knowledge of its existence and location. The data highlight that even though some young people were aware of its existence and whereabouts prior to accessing the service, others were less certain. This illustrates that even though there is awareness of the service through formal access pathways, such as school counsellors and GPs, and that youth can obtain information about the service through national and local website initiatives and in print and television media [20], there is a need for additional steps to be taken to make it more prominent in young people’s consciousness. One approach is to promote the service more prominently through social media, including on mobile-based technologies, given their popularity among young people. A second approach, launched after the completion of the current study’s fieldwork, is *e-headspace* (http://www.eheadspace.org.au), which increases young people’s access pathways by giving them opportunities to email, chat online or telephone a clinician. Indirect benefits of the service becoming more visible publicly are that it can improve access, an important feature of youth-friendliness [9,15,16], and can help counteract community stigma associated with youth depression [37] as something that is treatable. Another dimension of access is the geographical location of the service and its close proximity to public transport. Proximity to public transport is an important consideration because the service caters for young people who are more likely to be dependent on public transport than adults, who frequently have their own means of transport. However, the findings also show that those who do not live close to public transport themselves can still encounter difficulties, a finding that has also been highlighted in a study by Hodges et al. [9] of youth living in rural settings.

Third, it is evident that attempts to gain access to the service initially are met with, in some participants’ opinion, needless delays of several weeks while waiting for their first appointment. While the delays are relatively short in comparison to those at some other services, and the fact that they could have accessed the *headspace* GP if the need was great, the implication of delays is that they contribute to DUI, which may adversely affect the course of their depression [38,39]; early access to treatment increases the possibility of shorter illness duration and more favourable outcomes [40]. Arguably, the main issue is that delays will heighten the possibility that some young people may not persevere with their appointments. This is particularly the case for those who are embarrassed and self-conscious about help-seeking with professionals [4,14], or are reluctant to seek help even though they know or suspect they have a mental health problem such as depression [2,41]. A waiting period for appointments may be attributable to the high level of need in the community and limited availability of mental health services, reluctance to access GPs [4], and limited availability of alternative services for young people. It also may be attributable to insufficient government funding and a limited workforce [20], which restricts the availability of relevant services and, in turn, increases the demand for treatment in existing services. The implication of delay in gaining access is that the service can be conceived as less youth-friendly [9,16]. Furthermore, the delay and limited availability may lead some youth to seek assistance from adult mental health services, which are often ill-suited for this age group [13,17]. Indeed, as they are unlikely to gain access to adult services unless they are acutely ill and at high risk of self-harm, this can lead to disillusionment with services and a further increase in DUI.

Fourth, financial considerations impinge on youth access to, and ongoing involvement with, the service. It is evident that the low or no cost access to the service is an important consideration for some participants. The mixed method funding model (government and private insurance) operates as an access facilitator for the early part of treatment. In particular, government funding enables therapy to be offered ‘free’ to youth for a limited period (although some practitioners may impose a charge that is above the government rebate). This is made possible through the Commonwealth Government’s *Better Access to Mental Health Care Program*[36], which makes provision for fully subsidised treatment for up to 12 sessions of individual therapy per year (10 sessions from November 2011), and an additional six sessions in exceptional circumstances. Moreover, these limits are not applicable to treatment provided by a psychiatrist or a GP. The implication of this mixed funding model, including the fact that the service provides a low or no cost service, is that it enhances access to the service, reinforcing its perception as being youth-friendly [15], particularly to those from a lower socioeconomic background. This is an important matter for youth services located in or close to low socio-economic regions, as in the present study [42]. However, the reduction in the number of fully funded sessions from 12 to 10 is likely to have adverse implications for young people from these backgrounds.

Although it facilitates the early part of treatment, the service’s funding model subsequently operates as an access barrier, because it limits the number of treatment sessions that are available at no or minimal cost. This creates difficulties for young people with depression who may require more than the funded number of sessions but don’t meet the ‘exceptional circumstances’ requirement, especially those with limited financial means. With some exceptions, the consequence of this for those who need additional sessions is that they are compelled to pay for the service; revert to accessing other primary healthcare providers for treatment, such as GPs or counsellors who may not have the knowledge or skills to treat youth mental illness [17]; or withdraw prematurely from treatment and risk relapse, undermining the principle of youth-friendliness [15].

Overall, the findings of this study indicate that while young people’s experiences of accessing services share some similarities with adult consumers, such as the importance of establishing a trusting relationship with primary health care practitioners [43], there are important differences, including reliance on school counsellors and use of informal access pathways [3,4,9]. Furthermore, because of the age of young people they are less likely to have the same social networks and help-seeking skills as adult consumers, which may delay their help-seeking and increase the DUI [38,39].

### Limitations

Even though qualitative research presents an in-depth insight into the experience of young people with depression accessing this particular youth service, generalisability is not achieved from sample representativeness but from the concepts that are applicable to young people in a similar situation [44,45]. This is an important consideration because there are distinctions between this and other enhanced primary care services for young people, nationally and internationally. In particular, there is considerable heterogeneity in how *headspace* centres are set up and operate, and findings related to access to this particular service, which is located in a major metropolitan city, may not apply elsewhere. Furthermore, recruitment through key clinicians might have led to an atypical sample of engaged youth with a different experience to those who were less engaged with the service. Future research may benefit from having young people with depression who have disengaged prematurely from, or are not engaged with, the service.

## Conclusions

Youth depression is common and the longer the DUI the worse the outcome for the young person. Timely access to primary care services that address youth depression and related mental health problems is essential; however, personal and logistical factors affect this process. The findings of our IPA study have five key implications for young people’s help-seeking for depression and related mental health problems. First, school counsellors have a crucial role in facilitating young people’s access to youth services, and this important contribution should be maintained and supported. Likewise, primary care practitioners, such as GPs, school nurses and practice nurses, have an important role in providing timely access to care. Second, because youth may be reluctant initially to approach formal help-seeking pathways, it is important that there are ongoing initiatives to improve mental health literacy within the community, so that families, friends, school teachers and youth workers are better informed about how to recognise youth depression, as well as support them to access professional help. Third, the service’s public profile needs to be increased so that young people with mental health issues (especially those who are no longer in school and/or are reluctant to consult with their GP or seek assistance elsewhere), become more aware of the existence and youth-friendliness of the facility. Fourth, greater government funding is needed to increase the number of similar enhanced primary care services and to ensure they are located in settings that maximise access. Particular attention should be given to locating services in newer suburbs, often containing larger populations of young people and with poorer public transport infrastructure, in close proximity to shops and other amenities where youth are more likely to congregate. Furthermore, the government needs to review the funding model for this enhanced primary care service to ensure that young people, especially those from low socioeconomic backgrounds, are not prevented from having ongoing access to the service. Finally, research is needed to examine access pathways for young people with depression, with a focus on supporting those who are reluctant to seek help and with limited financial means, and to assess if there are differences between genders in the issues that they face in accessing services.

## Competing interests

The authors declare that they have no competing interests.

## Funding

The authors declare receipt of the following financial support for the study: The study was funded by a grant from Victoria University, Melbourne, Australia.

## Authors’ contributions

TVMcC was involved in securing funding for the study, had a major role in the design of the study, oversaw the data collection, carried out the data analysis, and had a major role in writing the paper. DIL had a major role in the design of the study, facilitated access to the service, contributed to the data analysis and writing the paper. Both authors have approved the final draft.

## Pre-publication history

The pre-publication history for this paper can be accessed here:

http://www.biomedcentral.com/1471-244X/12/96/prepub
